# HIV-1 Tat C phosphorylates VE-cadherin complex and increases human brain microvascular endothelial cell permeability

**DOI:** 10.1186/1471-2202-15-80

**Published:** 2014-06-26

**Authors:** Ritu Mishra, Sunit Kumar Singh

**Affiliations:** 1 Laboratory of Neurovirology and Inflammation Biology, CSIR-Centre for Cellular and Molecular Biology (CCMB), Uppal Road, Hyderabad 500007, India; 2 Laboratory of Human Molecular Virology & Immunology, Molecular Biology Unit, Faculty of Medicine, Institute of Medical Sciences (IMS), Banaras Hindu University (BHU), Varanasi 221005, India

**Keywords:** HIV-1 Tat C, HIV Neuropathogenesis, ROS, Adherens junction proteins, NOX2, NOX4, Blood brain barrier, Brain microvascular endothelial cells

## Abstract

**Background:**

Human brain microvascular endothelial cells (hBMVECs) are integral part of the blood brain barrier. Post-translational modifications of adherens junction proteins regulate the permeability of human brain microvascular endothelial cells. Pro-inflammatory signals can induce tyrosine phosphorylation of adherens junction proteins. The primary objective of this work is to provide a molecular model; how the HIV-1 Tat protein can compromise the BBB integrity and eventually lead to neurological consequences. We exposed hBMVECs to recombinant HIV-1 clade C Tat protein to study the effect of HIV-1 Tat C on permeability of hBMVECs. Trans-endothelial electrical resistance and fluorescent dye migration assay have been used to check the permeability of hBMVECs. DCFDA staining has been used for intracellular reactive oxygen species (ROS) detection. Western blotting has been used to study the expression levels and co-immunoprecipitation has been used to study the interactions among adherens junction proteins.

**Results:**

HIV-1 Tat C protein induced NOX2 and NOX4 expression level and increased intracellular ROS level. Redox-sensitive kinase; PYK2 activation led to increased tyrosine phosphorylation of VE-cadherin and β-catenin, leading to disruption of junctional assembly. The dissociation of tyrosine phosphatases VE-PTP and SHP2 from cadherin complex resulted into increased tyrosine phosphorylation of VE-cadherin and β-catenin in HIV-1 Tat C treated hBMVECs.

**Conclusion:**

Unrestricted phosphorylation of junctional proteins in hBMVECs, in response to HIV-1 Tat C protein; leads to the disruption of junctional complexes and increased endothelial permeability.

## Background

HIV enters the central nervous system (CNS) by crossing the blood brain barrier (BBB) during early stages of HIV infection through infected blood cells [1,2]. HIV infected cells secrete the Tat protein extracellularly [3] and affect the functions of human brain microvascular endothelial cells (hBMVECs) [4]. The hBMVECs form a selective barrier between peripheral circulation and the CNS. The barrier properties of hBMVECs are principally imparted by adherens junction proteins (AJPs) and tight junction proteins (TJPs) [5]. VE-cadherin and catenin play major role in stabilization of adherens junctions (AJs) and barrier integrity [6]. Cadherin complex is composed of vascular endothelial-cadherin (VE-cadherin), bound to β-catenin or plakoglobin, which in turn binds to α-catenin; vinculin, α-actinin, ZO-1 and actin [7]. The tyrosine phosphorylation of VE-cadherin complex is critical for the integrity of the AJs and endothelial permeability [8,9].

VE-PTP is a tyrosine phosphatase, transmembrane binding partner of VE-cadherin. It connects with VE-cadherin through an extracellular domain and maintains the unphosphorylated state of VE-cadherin [10]. Another phosphatase; SHP2 is a ubiquitously expressed non-receptor PTP, consisting of two N-terminal tandem SH2 domains and a catalytic phosphatase (PTP) domain and a C-terminal tail with two tyrosine residues [11]. Ukropec *et al*. [12]; reported SHP2 as a component of VE-cadherin complex, which regulates the tyrosine phosphorylation of both, β-catenin [12] and VE-cadherin [13].

Phosphorylated states of the VE-cadherin and β-catenin regulate their interactions, and control the adhesion forces [14]. VE-cadherin has many putative phospho-tyrosine sites, but Y658, Y685, and Y731 phosphorylation are reported to be important for maintaining the barrier integrity [15]. PYK2 is a redox-sensitive tyrosine kinase and gets activated by NADPH mediated ROS generation [16]. Tyrosine phosphorylation of β-catenin is induced by PYK2 (proline rich kinase 2) [17] and dephosphorylated by SHP2 [18].

Pro-inflammatory factors affect the phosphorylated state of VE-cadherin complex and disrupt the barrier integrity [19,20]. In this study, we have selected HIV-1 Tat C to study the effect on endothelial permeability, because HIV-1 clade C alone is responsible for more than 56% of global HIV infections. HIV-1 clade C infections are prevalent in South-east Asian countries including India [21]. We have examined the effects of the HIV-1 Tat C protein on redox-sensitive kinase PYK2 and tyrosine phosphatases of VE-cadherin complex (VE-PTP and SHP2) and their impact on tyrosine phosphorylation of VE-cadherin, β-catenin and permeability of hBMVECs.

## Methods

### Cell culture

Primary hBMVECs were obtained from Dr. Joan Berman, department of pathology, Albert Einstein College of Medicine, New York as a kind gift. hBMVECs were cultured as described previously [22]. For all hBMVECs cultures, the culture flasks were coated with 0.2% bovine gelatin solution. CEM-GFP cells (NIH-AIDS Reagent Programme) were used for transactivation assay to check the biological activity of recombinant HIV-1 Tat C protein. CEM-GFP cells were grown in RPMI 1640 (Gibco) supplemented with 10% fetal bovine serum (Gibco), 2 mM glutamine, 100 units of penicillin/streptomycin/ml. Cell cultures were maintained at 37°C and constant supply of 5% CO_2_ in a humidified incubator.

### Expression and purification of HIV-1 Tat C protein

HIV-1 Tat C protein has been expressed and purified as per our standardized protocol, described elsewhere in detail [23]. Immunoconfirmation of recombinant HIV-1 Tat C protein was done by western blot analysis using anti-Tat antibody (NIH AIDS Research and Reference Reagent Program). Endotoxin level of purified recombinant HIV-1 Tat C protein was measured by Limulus Amebocyte Lysate (LAL) assay (Lonza) as per manufacturer’s protocol. The level of endotoxin was found in range of 0.04 EU/μg of purified protein; which was much below the acceptable limit set by international standards. Transcriptional activity of recombinant HIV-1 Tat C protein was checked by transactivation assay as described previously [23]. CEM-GFP cells are T-cell lines, carrying a stably integrated GFP gene under the control of HIV-1 subtype-B LTR. Purified HIV-1 Tat C protein (5 μg) was transfected in CEM-GFP cells, with proteo-juice protein transfection reagent (Novagen). GFP expressions were visualized with fluorescence microscope (Axio Observer-A1, Carl Zeiss, Germany). All protocols were approved by the Centre for Cellular and Molecular Biology (CCMB), Hyderabad Institutional Biosafety Committee.

### HIV-1 Tat C treatment on human brain microvascular endothelial cells

hBMVECs were grown as confluent monolayer and exposed with HIV-1 Tat C protein at various doses (100 ng/ml, 200 ng/ml, 500 ng/ml, and 1 μg/ml) in serum-free M199 media. Control cells were treated with Tat buffer (30 mM phosphate buffer, pH 6.8 supplemented with 1 mM DTT). BMVECs have been exposed to Tat C at different concentration ranges due to its closeness to reported serum levels and hBMVECs would normally encounter such concentrations of Tat in NeuroAIDS patients [3,24,25]. hBMVECs were harvested for RNA and protein sample preparation after 12 hours of HIV-1 Tat C protein treatments.

### Western blot analysis

Cell lysis were done in RIPA buffer (150 mM NaCl, 50 mM Tris.HCL pH 7.5, 1% NP-40, 0.5% sodium doxycholate, 0.1% SDS and 1× protease inhibitor cocktail). Estimation of protein concentrations was done by Bradford assay (Bio-Rad). Proteins samples were boiled in SDS-loading buffer and run on 12% SDS-gel, transferred on PVDF membrane (Millipore) at 100 V for 2 hrs. Primary antibody incubations were given overnight at 4°C with dilutions (1:1000). HRP conjugated secondary antibody were applied for 1 hour at room temperature and developed by using Super Signal developing reagent (Pierce, Thermo Scientific). Antibodies against p-Y-731-VE-cadherin (Invitrogen), VE-cadherin, anti-β-tubulin, NOX2 and NOX4 (Abcam), β-catenin (Santacruz biotechnology), PYK2 (Cell signaling technology), SHP2 (Sigma) have been used in the study. VE-PTP antibody was provided as a kind gift by Astrid F. Nottebaum, Max Planck Institute of Molecular Medicine, Muenster, Germany.

### Transendothelial Electrical Resistance (TEER) Assay

EVOM2 (World precision Instruments) was used to measure electrical resistance across the endothelial monolayer. PTFE membrane inserts (12 well plate) (Corning Life sciences) consisting of 3.0 μm pore sizes were used in the permeability assays. 0.2% bovine gelatin coating was done on insert membrane before cell seeding for better adherence of hBMVECs. Cell seeding density was 2 × 10^4^ in 500 μl of complete M199 media. To check the development of junctional integrity, TEER values were checked on regular intervals until reached at consistent resistance in endothelial cells. After attaining the optimal confluency, the hBMVECs were treated with different doses of HIV-1 Tat C protein. TEER of hBMVECs was measured after 12 hrs of HIV-1 Tat C protein exposure. The resistance of empty insert membranes, without cell seeding was deducted from recorded TEER values.

### Fluorescein migration assay

The fluorescein migration assay was performed as described previously elsewhere [22]. hBMVECs were seeded on membrane inserts in similar way as for TEER assay. After treatment with different doses of Tat C proteins, cells were washed with HBSS buffer and incubated with 0.005% sodium fluorescein dye (SRL # 064738, mol weight- 376.28) on upper chamber of the insert membrane. hBMVECs were incubated with sodium fluorescein dye for 30 minutes at 37°C, followed by collection of 100 μl of the flowthrough from lower chambers. Fluorescence was measured at 480/530 nm wavelength by using fluorimeter (Infinite M200, Tecon).

### Co-immunoprecipitation of VE-cadherin/VE-PTP/β-catenin and SHP2

Cells were pelleted after 12 hours of HIV-1 Tat C treatment (500 ng/ml), washed with cold PBS and lysed in NP-40 mild lysis buffer (NP-40 1%, Tris–HCl 50 mM, NaCL 150 mM, NaF 1 mM, PMSF 2 mM, Sodium orthovanadate 1 mM, Sodium pyrophosphate 10 mM, Protease inhibiter cocktail 1×). Equal amounts of protein samples were incubated with VE-cadherin antibody overnight at 4°C. These samples were incubated with protein-G-sepharose beads for 3–4 hours followed by centrifugation. The immunoprecipitated samples along with beads were washed three times with lysis buffer and finally eluted by boiling in SDS-loading buffer. The samples were run on 12/% SDS-Page gel and probed with respective antibodies.

### Intracellular Reactive Oxygen Species (ROS) detection by Microscopy

hBMVECs were grown in 6 well culture dishes till confluency. HIV-1 Tat treatment was given for 12 hours at the different doses as described earlier. Cells were washed with HBSS and treated with final concentration of 10 μM of cell permeant fluorogenic dye 2’ 7’ dichlorofluorescein diacetate (DCFDA) for 15 minutes at 37°C in incubator chamber. After 15 minutes of incubation, cells were washed and fluorescence was visualized under fluorescence microscope (Axio Observer-A1, Carl Zeiss, and Germany).

### Inhibition experiments

Chemical inhibiter against PYK2 (PF-431396) (Tocris Bioscience) has been used to inhibit the kinase activity. hBMVECs were cultured till confluency and treated with 22 nM PF-431396. Cells treated with equal volume of DMSO have been taken as control. Diphenyleneiodonium chloride (DPI) (Sigma Aldrich) dissolved in DMSO has been used as ROS scavenger, at 100 nM concentration. hBMVECs were harvested for lysate preparation after 12 hours of treatment.

### Statistical analysis

Results have been expressed as the mean and standard error of the mean. Level of significance in treated group versus untreated group has been measured by using Student’s t test. P-values < 0.05 have been taken as significant in one tailed array.

## Results

### HIV-1 Tat mediated phosphorylation of adherens junction protein (VE-cadherin and β-catenin)

**Figure 1:**
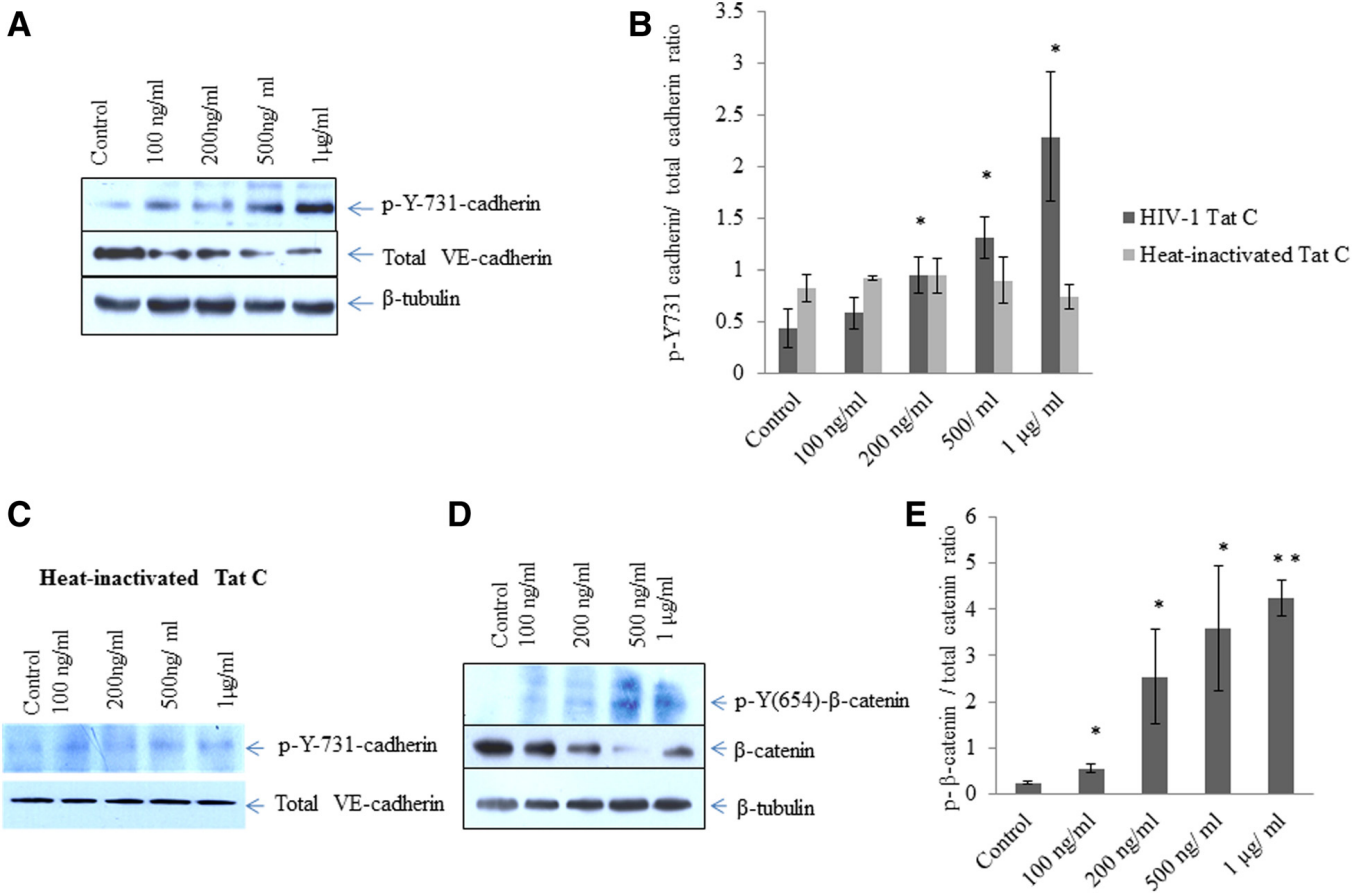
**HIV-1 Tat C mediated phosphorylation of adherens junction proteins (VE-cadherin and β-catenin). (A)** Western blot analysis to check the effect of HIV-1 Tat C protein in hBMVECs, showing a dose dependent increase in tyrosine phosphorylation of VE-cadherin (p-Y-731). **(B)** The graph showing the result of densitometry for western blot images showing significantly induced tyrosine phosphorylation (p-Y-731) of VE-cadherin upon HIV-1 Tat/ heat inactivated Tat treatment. Quantitation of image densities have been done by ImageJ software normalized with β-tubulin. **(C)** Western blot analysis showing the insignificant effect of heat inactivated HIV-1 Tat C protein on tyrosine phosphorylation (p-Y-731) of VE-cadherin **(D)** Western blot analysis to show dose dependent increase in tyrosine phosphorylation of β-catenin (p-Y-654) in hBMVECs exposed to HIV-1 Tat C protein. **(E)** Densitometry analysis of image density to show change in tyrosine phosphorylation (p-Y-654) of β-catenin. Image densities have been normalized with β-tubulin. All the experiments have been performed in three independent biological sets and results are presented as mean ± S.E., *above bars are representing the p value ≤0.05 as level of significance, n = 3. (*for p value ≤0.05, **for p value ≤0.005).

We investigated the tyrosine phosphorylation of VE-cadherin and β-catenin in hBMVECs treated with increasing doses from 100 ng/ml to 1 μg/ml of HIV-1 Tat C. Both the adherens junction proteins (AJPs) (VE-cadherin and β-catenin) were significantly phosphorylated up to 4 folds at their respective tyrosine positions in dose dependent manner (Figure 1). We checked the tyrosine phosphorylation (Y-731) of VE-cadherin in hBMVECs exposed to heat inactivated Tat C and couldn’t find any substantial alteration in VE-cadherin phosphorylation (Figure 1B, C).

### Downregulation and dissociation of VE-PTP and SHP2 from VE-cadherin complex in HIV-1 Tat treated human BMVECs

**Figure 2:**
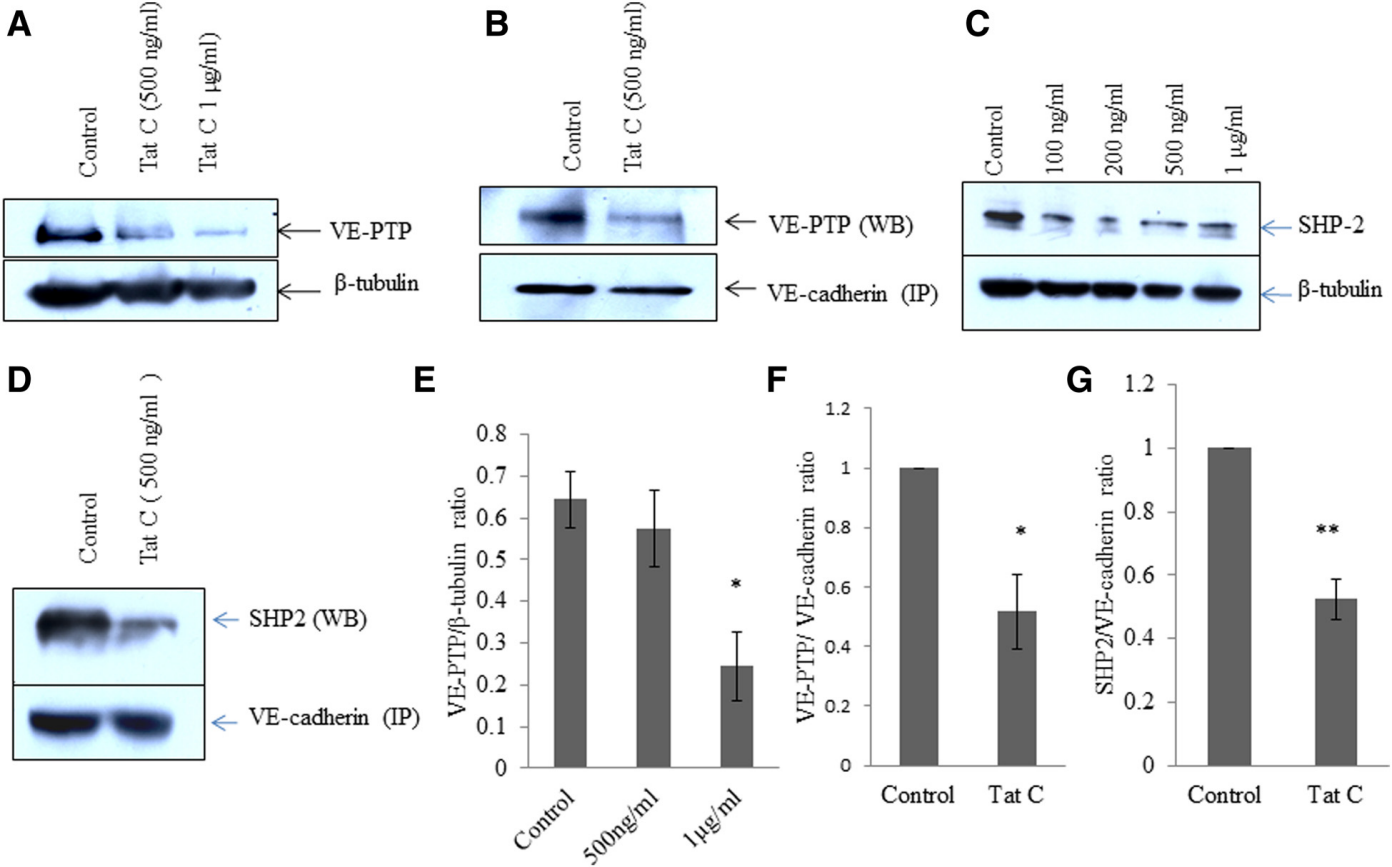
**Downregulation and dissociation of VE-PTP and SHP2 from VE-cadherin in HIV-1 Tat C treated BMVECs. (A)** Downregulation of VE-PTP expression in HIV-1 Tat C treated hBMVECs. HIV-1 Tat C treatment has been given for 12 hours on hBMVECs and western blot analysis was done by using anti VE-PTP antibody to show decrease in expression level of phosphatase VE-PTP. **(B)** Immnunoblot images of co-immunoprecipitaion of VE-PTP along with VE-cadherin complex showing the decreased association of VE-PTP with VE-cadherin. Same blots have been re-probed with VE-cadherin antibody to exhibit equal loading of pulled down cadherin complex. Monoclonal VE-cadherin antibody has been used for pull down of VE-cadherin complex by using NP-40 mild lysis buffer in cold conditions. **(C)** Compared to control hBMVECs (buffer treated), HIV-1 Tat C treated hBMVECs are showing a dose-dependent decrease in SHP2 expression levels. **(D)** SHP2 dissociation from cadherin complex has been shown by immunoblotting against SHP2 in pulled down cadherin complex by using anti VE-cadherin antibody. **(E)** The densitometry graph is showing almost 60–70% decrease in VE-PTP expression at higher doses of HIV-1 Tat C treatment. **(F)** Quantitation of image density by ImageJ software normalized by VE-cadherin image density. Pull-down experiment has been repeated three times and results are shown as mean ± S.E. (*p value ≤0.05). **(G)** Quantitation of image density of SHP2 showing a significant dissociation (**p value ≤0.005) from cadherin complex, normalized with amount of VE-cadherin pull down. All the results shown here are representative of three independently repeated experiments and shown as mean ± S.E.

VE-PTPs are essentially associated with VE-cadherin and perform the function of keeping VE-cadherin in dephosphorylated state in quiescent cells [10]. We observed a dose dependent increase in tyrosine phosphorylation of VE-cadherin (Y731 position) in HIV-1 Tat C exposed hBMVECs (Figure 1A, B). We further investigated the levels of VE-PTP and their association with VE-cadherin in HIV-1 Tat exposed hBMVECs. We observed decreased expression of total VE-PTP (Figure 2A, E), along with the significant dissociation (p ≤ .05) of VE-PTP from VE-cadherin complex in HIV-1 Tat treated hBMVECs (Figure 2B, F). SHP2, a non-receptor protein-tyrosine phosphatase co-precipitates with VE-cadherin complex in basal state of cells [12]. SHP2 phosphatase maintains the integrity of VE-cadherin complex, by dephosphorylating VE-cadherin-associated β-catenin as well as VE-Cadherin [13,18]. SHP2 expression levels decreased significantly (p ≤0.005) along with increasing dose of HIV-1 Tat (Figure 2C). The pull down experiment with unit amount of VE-cadherin complex by using monoclonal VE-cadherin antibody has shown significant reduction (p ≤0.005) in SHP2 association with VE-cadherin complex (Figure 2D, G). We observed that both the tyrosine phosphatases regulating the tyrosine phosphorylation, get dissociated from the VE-cadherin complex in HIV-1 Tat C exposed hBMVECs.

### HIV-1 Tat dissociates β-catenin from the VE-cadherin/β-catenin complex and induces endothelial permeability

**Figure 3:**
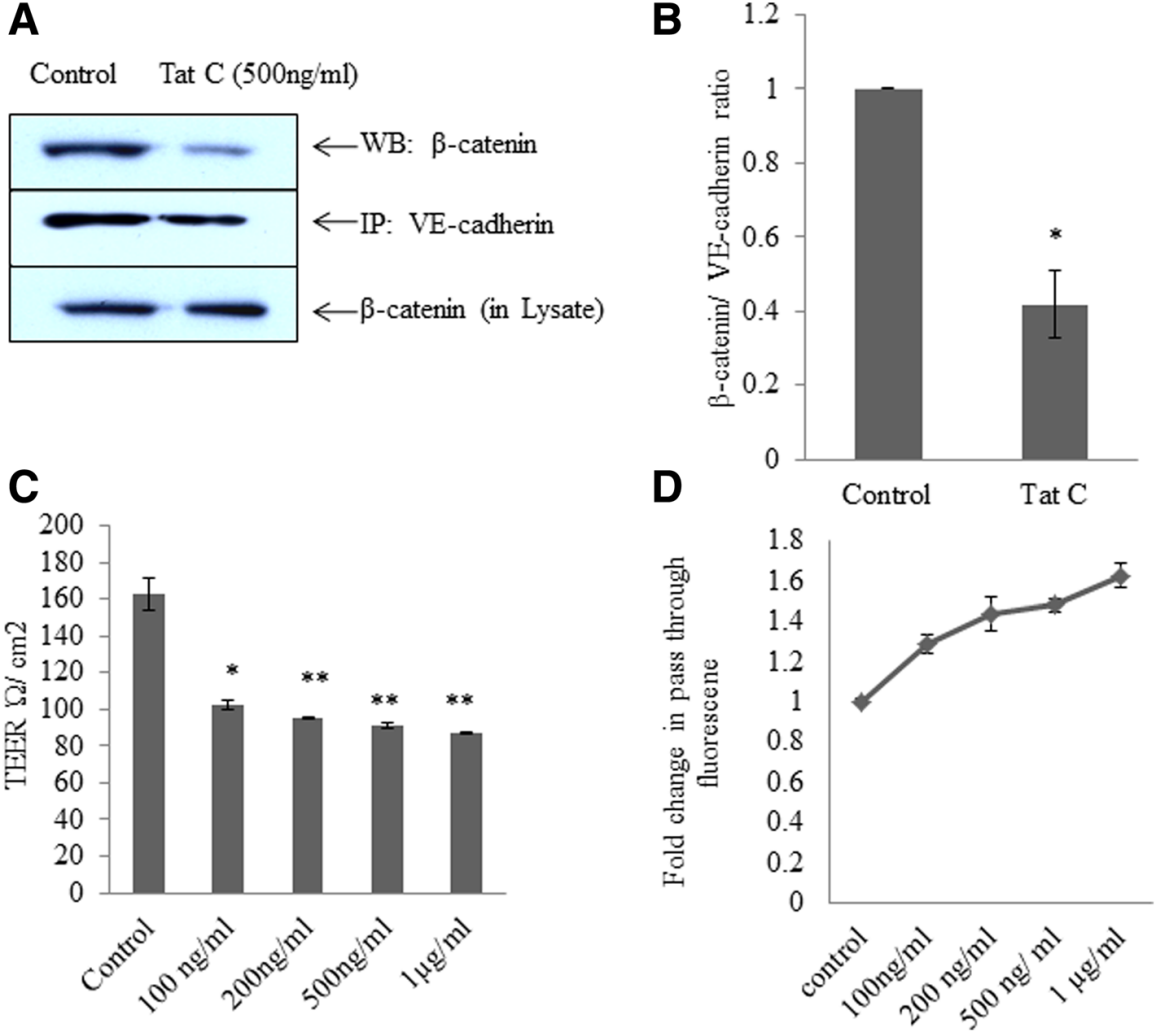
**HIV-1 Tat C dissociates β-catenin from the VE-cadherin/β-catenin complex and induces endothelial permeability.** VE-cadherin complex were pull down from control as well as HIV-1 Tat C treated hBMVECs to examine the phosphorylation induced dissociation of β-catenin from VE-cadherin. **(A)** Coimmunoprecipetation of β-catenin with cadherin complex, by using monoclonal anti VE-cadherin antibody, showing a significantly decreased association of β-catenin with VE-cadherin complex in HIV-1 Tat C treated hBMVECs. **(B)** A significant change in association of β-catenin with VE-cadherin in HIV-1 Tat C treated hBMVECs, shown by bar diagram. The bars are representing mean ± S.E. from three independently repeated experiments. **(C)** The graph showing a dose dependent decrease in TEER values across the trans-well insert membrane, in HIV-1 Tat C treated hBMVECs as compared to buffer treated control cells. **(D)** Sodium fluorescein dye migration assay, showing a dose dependent increase in amount of flow through of fluorescent dye across the trans-well insert membrane, due to increased permeability of hBMVECs exposed to increasing dose of HIV-1 Tat C protein.

HIV-1 Tat induced tyrosine phosphorylation of β-catenin occurs in dose dependent manner (Figures 1D, E). SHP2 and VE-PTP expression also got downregulated and dissociated from VE-cadherin complex after HIV-1 Tat treatment (500 ng/ml) in hBMVECs, leaving VE-cadherin complex prone to the phopshorylation (Figure 2). Co-immunoprecipitation of the cadherin complex by monoclonal antibody of VE-cadherin showed 60% reduction (P ≤ 0.05) in the association of β-catenin with VE-cadherin in HIV-1 Tat treated hBMVECs (Figure 3A, B). Endothelial cell permeability, assessed by TEER and sodium fluorescein migration assay showed an elevated permeability in HIV-1 Tat, treated hBMVECs (Figure 3C, D). Compared to control cells, HIV-1 Tat treated hBMVECs, has shown 40–50% decrease in TEER values, however sodium fluorescein migration assay has shown about 60–70% increase in endothelial cell permeability in trans-well membrane experiments (Figure 3C, D).

### HIV-1 Tat upregulates NADPH oxidase (NOX2 and NOX4) expression and increases ROS production in human BMVECs

**Figure 4:**
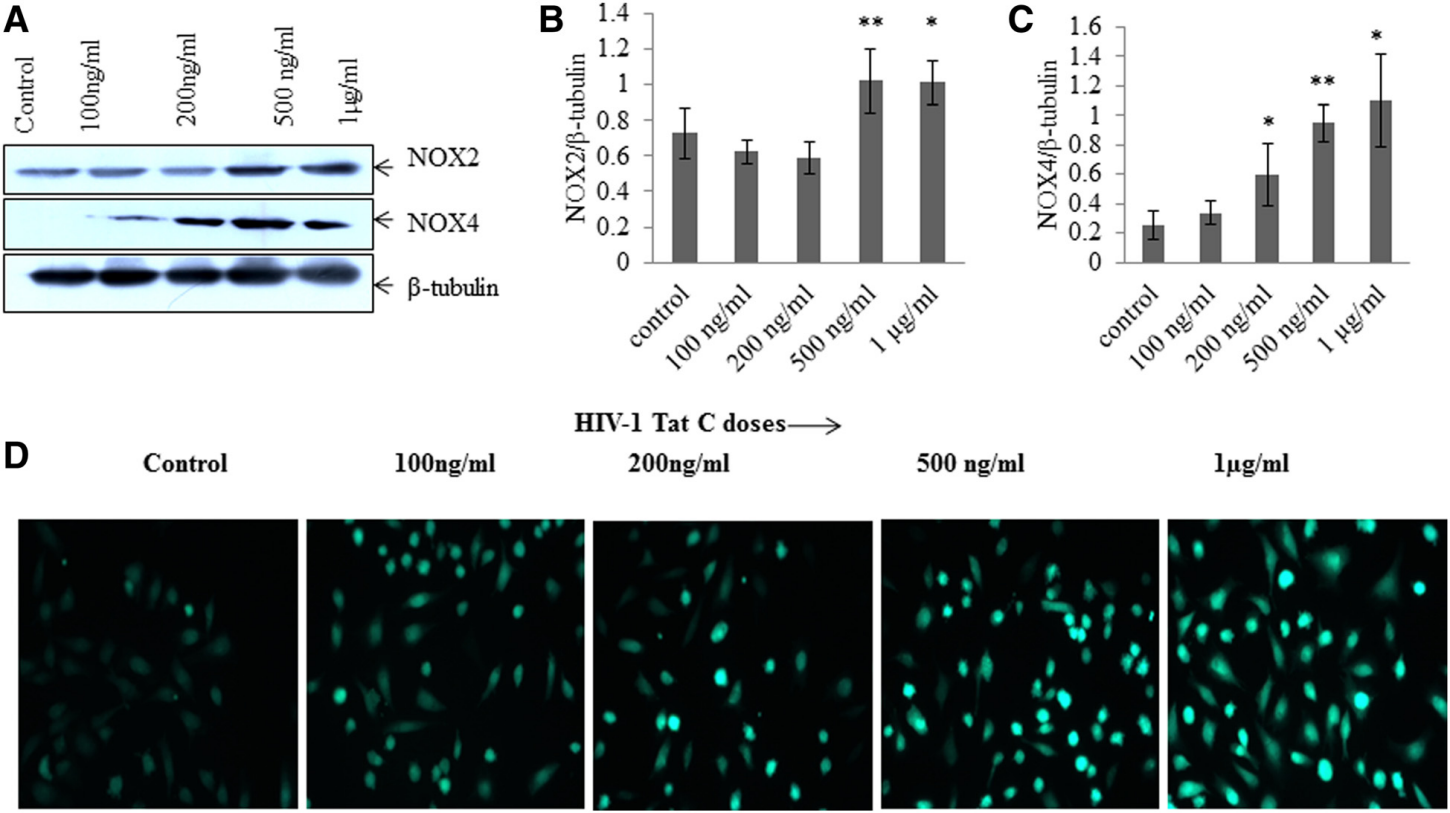
**HIV-1 Tat C upregulates NOX2 and NOX4 expression and increases ROS production in hBMVECs. (A)** Western blot analysis to monitor the changes in expression levels of NOX2 and NOX4 in hBMVECs exposed to HIV-1 Tat C protein. The level of expression of both the NOX proteins increased after HIV-1 Tat C treatment, and led to enhanced ROS production by hBMVECs in a dose–dependent manner. **(B, C)** Densitometry for western images for NOX2 and NOX4 normalized with image density of β-tubulin. The graphs are representative of three independent experiments and results are shown as mean ± S.E. The levels of significance in the changes of the gene expression; compared to control groups, the p values ≤0.05 are indicated as *above the bar, (*for p value ≤0.05, **for p value ≤0.005). **(D)** The figure showing fluorescence microscopic images of intracellular ROS level in hBMVECs detected via DCFDA staining. Increasing doses of HIV-1 Tat C treatments are showing increase in ROS level in linear fashion. hBMVECs were treated with HIV-1 Tat C protein, washed with HBSS and incubated with DCFDA reagent (10 μM final concentration) for 15 minutes, washed and visualized under microscope. Images were captured by Axio Observer-A1 at 20 × magnification with GFP filters.

NADPH oxidases regulate ROS production; which can affect the PTK (protein tyrosine kinase) and PTP (protein tyrosine phosphates) balance in cells [26]. Intracellular ROS level can regulate PYK2 activation and might play role in VE-PTP dissociation from cadherin complex [16]. We checked the expression level of NOX2 and NOX4 and overall intracellular ROS production by DCFDA staining. We observed the significant (p <0.005) induction in the expression levels of NOX2 and NOX4 in HIV-1 Tat C exposed hBMVECs (Figure 4A-C) and increase in the ROS production in dose dependent manner (Figure 4D).

### HIV-1 Tat activates PYK-2 in dose dependent manner

**Figure 5:**
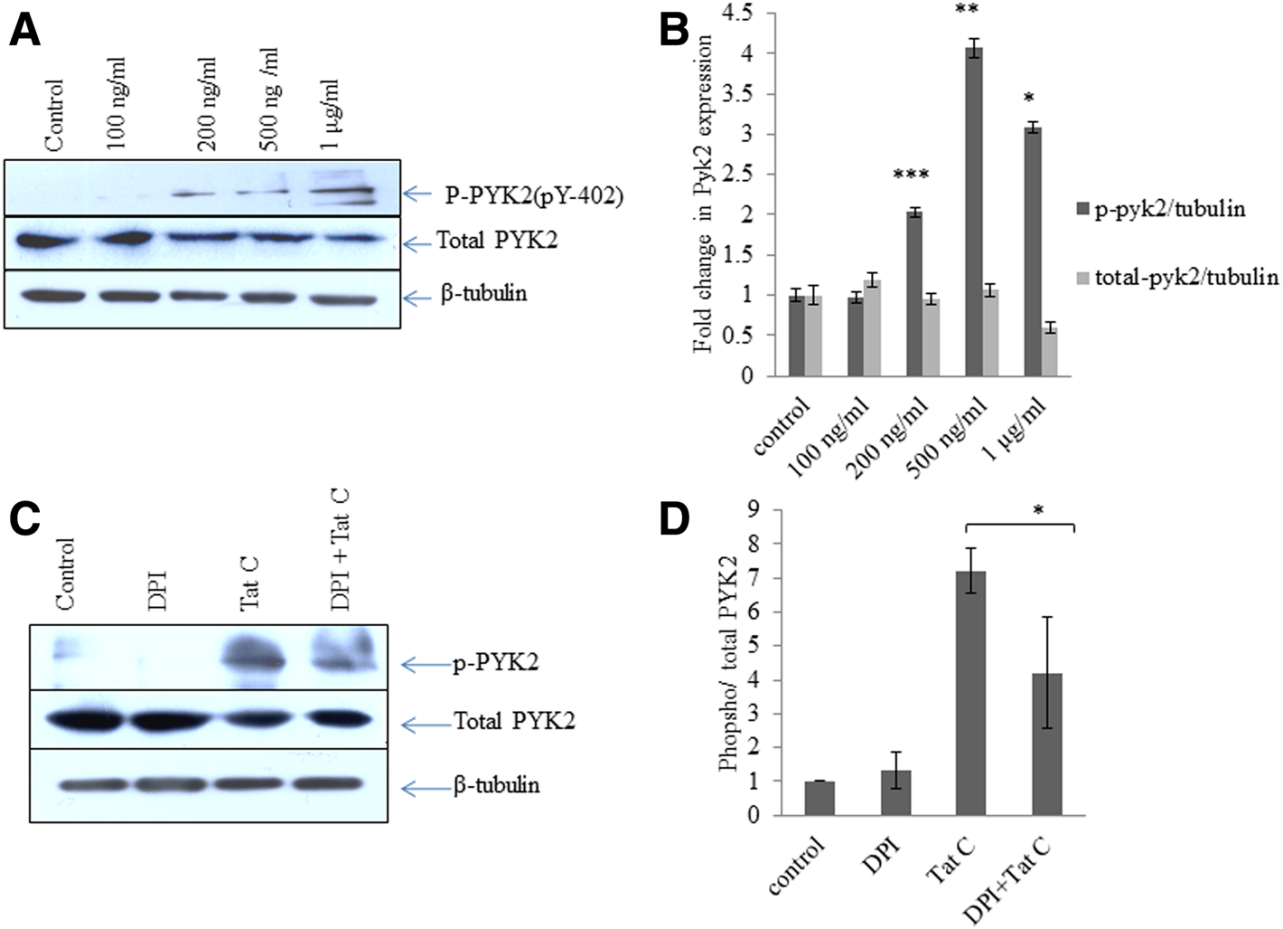
**HIV-1 Tat C activates PYK-2 in dose dependent manner. (A)** Western blot images showing a dose dependent increase in activity of PYK2 as increased phosphorylated form of PYK2 at Y-402 position in HIV-1 Tat C treated hBMVECs, as compared to control (buffer treated hBMVECs). **(B)** Densitometry of western blot images, done by ImageJ software to show quantitative changes in phosphorylation of PYK2 at different doses of HIV-1 Tat C treatment. Higher doses of HIV-1 Tat C protein significantly activated PYK2 (***shown for p value ≤0.0005) in three biological repeated experiments and results are shown as mean ± S.E. **(C)** Western blot analysis for phosphorylated PYK2 showing effect of ROS scavenger (DPI) on PYK2 activation. **(D)** The graph bars are showing densitometry to show average change in phosphorylated PYK2 after DPI treatment. P value ≤0.05 shown as *to show the significance level of change between Tat C versus DPI + Tat C treated group.

Proline-rich tyrosine kinase 2 (PYK2) is a non-receptor tyrosine kinase, belongs to the focal adhesion kinase (FAK) family [27]. HIV-1 Tat C exposed hBMVECs showed a dose dependent increase in expression level of NOX2 and NOX4 and levels of intracellular ROS. We tested the level of phospho-PYK2 in HIV-1 Tat C treated hBMVECs to delineate the role of PYK2 activation after Tat C treatment and to further find the role of PYK2 activation in tyrosine phosphorylation of β-catenin and VE-cadherin. A significant increase in the levels of phospho-PYK2 (at Y-402) was observed in hBMVECs (p ≤ .005) with increasing dose of HIV-1 Tat C protein (Figure 5A, B). Application of 100nM DPI; as ROS scavenger resulted into decreased PYK2 activation/phosphorylation (Figure 5C). ROS scavenging helped in combating the effect of HIV-1 Tat C on PYK2 activation (Figure 5C, D).

### ROS scavenger (DPI) mediated rescue of SHP2 expression and downregulation in phosphorylation of β-catenin

**Figure 6:**
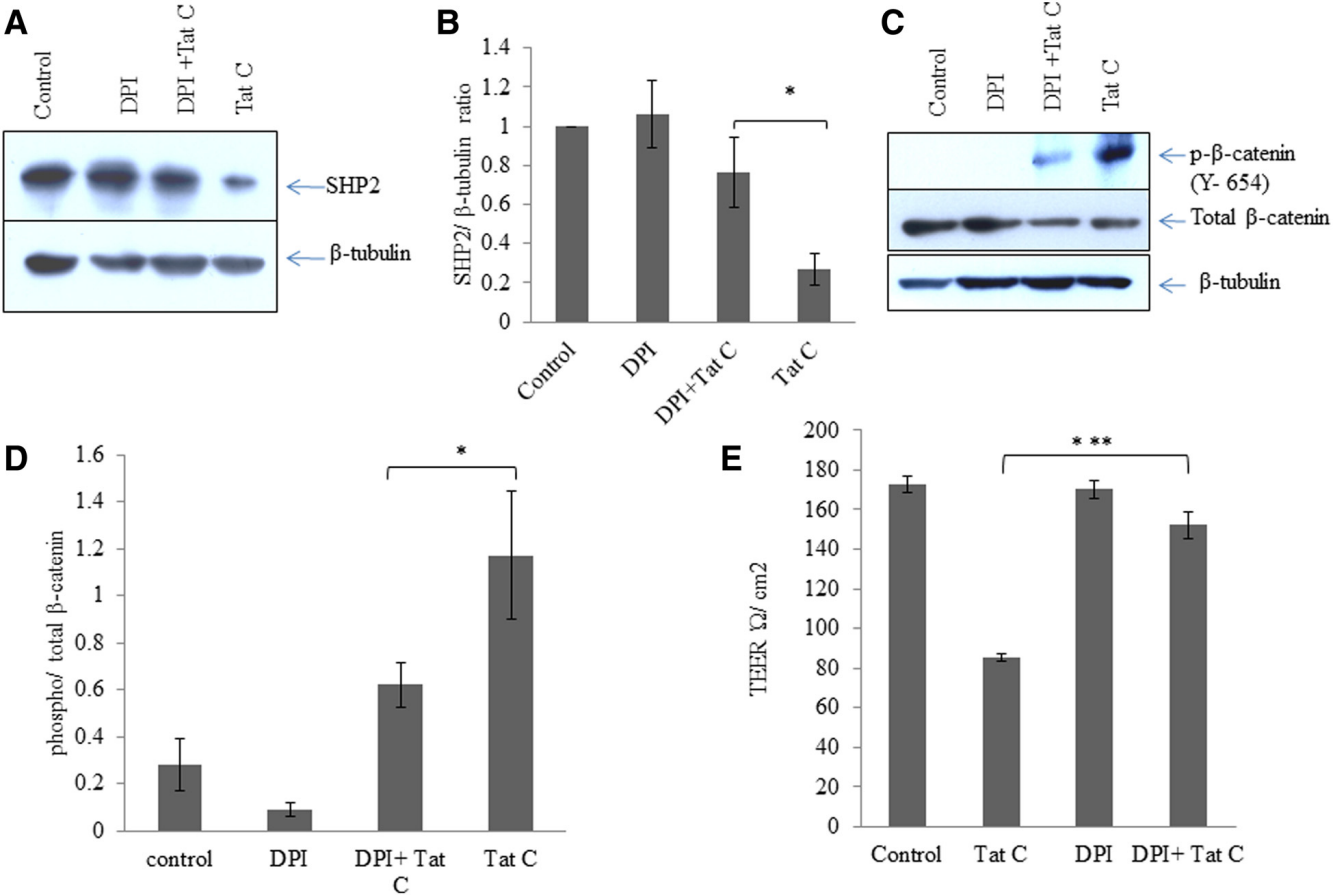
**ROS scavenger (DPI) mediated rescue of SHP2 expression and downregulation in phosphorylation of β-catenin. (A)** Western blot analysis for SHP2 expression level after DPI treatment and DPI treatment followed by HIV-1 Tat C treatment. DPI treatment shows significant potential for checking the HIV-1 Tat C mediated downregulation of SHP2 expression. **(B)** The graph bars are showing average change in SHP2 expression normalized by β-tubulin. Image densitometry analysis has been done by ImageJ software. **(C)** Western blot analysis showing effect of ROS inhibition on tyrosine phosphorylation of β-catenin. **(D)** The graph bars are representative of 3 independent experiments showing average change in tyrosine phosphorylation of β-catenin in DPI treated hBMVECs. P-value ≤0.05 has been considered as significant and shown as *between DPI + Tat C versus HIV-1 Tat C treated hBMVECs. **(E)** Change in TEER (Ώ/cm2) values after DPI treatment. The graph bars showing average change in endothelial permeability after scavenging ROS by DPI. All the experiments have been performed three times independently and data shown as mean ± S.E. P value ≤0.0005 shown as ***asterisk to indicate the level of significance between Tat C treated versus DPI + Tat C treated hBMVECs.

Redox-sensitive PYK2 activation and phosphorylation of β-catenin leads to conformational disorganization of cadherin complex in endothelial cells [28]. We exposed hBMVECs with ROS scavenger DPI and studied the effect of blocking ROS generation on PYK2 activation, SHP2 expression, phosphorylation of β-catenin, and endothelial permeability. We observed a sustained expression level of SHP2 in DPI treated hBMVECs (Figure 6A, B). Interestingly, DPI treatment helped in rescuing of SHP2 expression even in Tat treated hBMVECs (Figure 6A, B), which suggested the potential of blocking the ROS on kinase/phosphatase balance in hBMVECs. We checked the tyrosine phosphorylated state of the β-catenin in DPI treated hBMVECs. DPI treatment alone showed inhibition of the phosphorylation of β-catenin (Figure 6C, D) compared to highly phosphorylated state (Y-654) of β-catenin in HIV-1 Tat exposed hBMVECs. DPI treatment followed by Tat treatment resulted into decreased tyrosine phosphorylation of β-catenin. Tat induced tyrosine phosphorylation of β-catenin mediated by ROS; resulted into imbalance of kinases/phosphatases. The ROS scavenging helped in ameliorating the effect of HIV-1 Tat protein on perturbed phosphorylation of β-catenin. We observed that ROS inhibition by DPI treatment has significantly helped in mitigating the effect of HIV-1 Tat C protein on permeability of hBMVECs (p ≤0.005) (Figure 6E).

### PYK2 inhibiter abrogates the phosphorylation of β-catenin and rescues endothelial permeability

**Figure 7:**
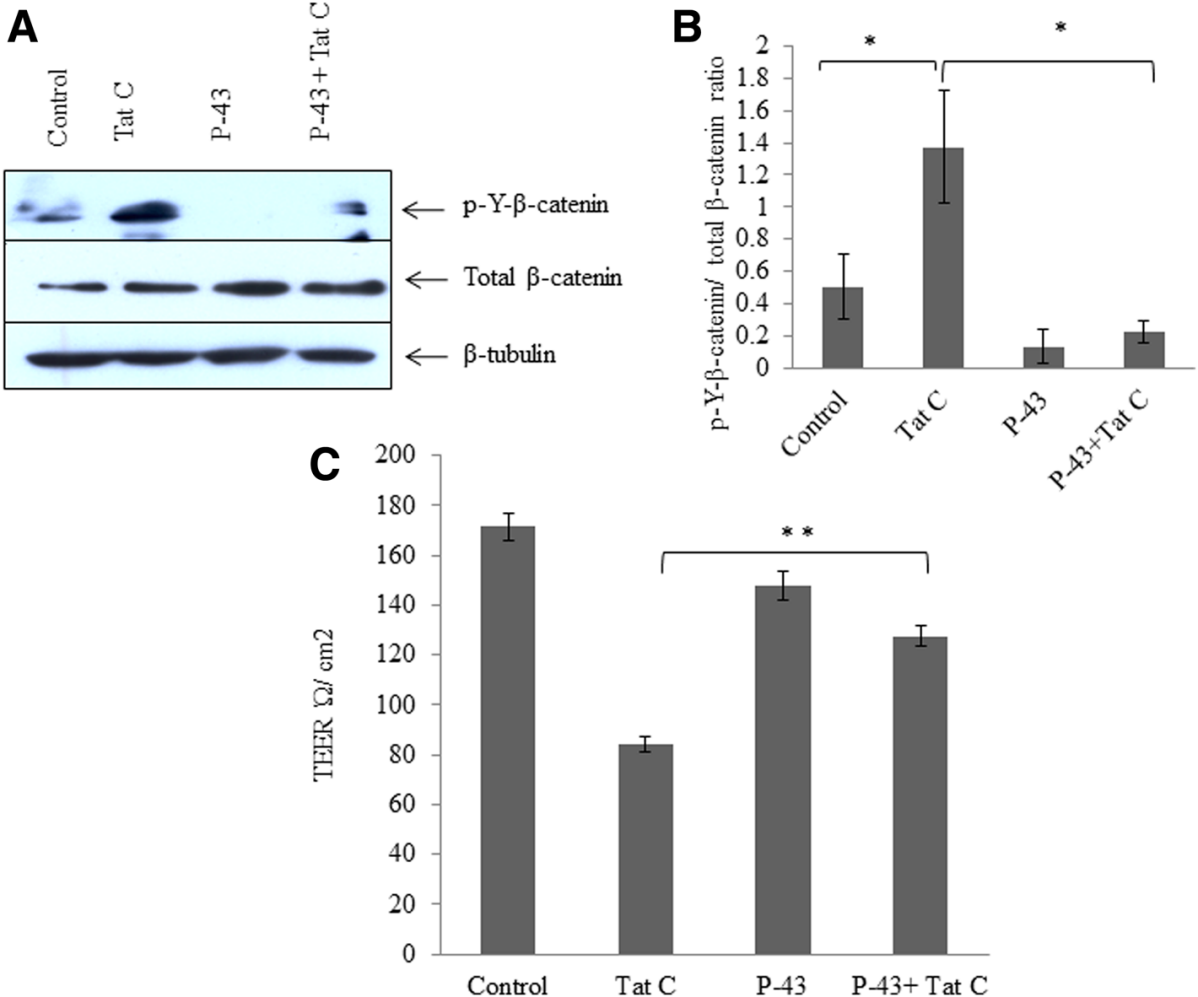
**PYK2 inhibiter abrogates the phosphorylation of β-catenin and rescues endothelial permeability. (A)** Tyrosine phosphorylation of β-catenin demonstrated after PYK2 inhibition by applying chemical inhibiter PF-431396. PYK2 inhibition significantly abrogated the tyrosine phosphorylation of β-catenin even in HIV-1 Tat C exposed hBMVECs. Cells were treated with PF-431396 followed by treatment of HIV-1 Tat C protein and treated cells were harvested after 12 hours for western blot analysis. **(B)** Densitometry analysis showing the significantly diminished tyrosine phosphorylation of β-catenin in PF-431396 treated cells. All the experiments have been repeated as three independent biological sets and results are shown as mean ± S.E. (*p value ≤0.05). **(C)** The graph bars are showing consequences of inhibiting PYK2 kinase activity on endothelial permeability. PYK2 inhibiter provide significant rescuing of endothelial permeability as compared to HIV-1 Tat C treated human BMVECs (**p value ≤0.005). Results are shown as mean ± S.E of three biological independent experiments.

hBMVECs were treated with PYK2 inhibiter PF-431396 (Inhibiter of kinase activity) (Tocris Bioscience) and checked for tyrosine phosphorylation of β-catenin. Significant reduction in tyrosine phosphorylation of β-catenin resulted into an accumulation of total β-catenin (Figure 7A, B). No significant effect on tyrosine phosphorylation of β-catenin was observed in hBMVECs treated with PYK2 inhibiter (PF-431396), followed by HIV-1 Tat treatment. The level of total β-catenin was comparable to PF-431396 treated hBMVECs (Figure 7A, B). The effect of PF-431396 was also investigated on the permeability of hBMVECs. The inhibition of PYK2 significantly abrogated the effect of HIV-1 Tat and maintained the endothelial permeability (p ≤0.005) (Figure 7C).

## Discussion

Neurological consequences still persist in the era of HAART therapy, where viral load has been effectively controlled in infected patients. However HIV-1 protein such as Tat has been detected in the CSF of patients kept on anti-retroviral therapy and show effectively controlled viremia [29]. Therefore, we have focused on the bystander effect of HIV-1 Tat protein on human brain microvascular endothelial cells. In brain microvascular endothelial cells, the levels of VE-cadherin regulate the permeability of endothelial cells [9]. Vascular endothelial cells are connected by homophilic adhesion through AJPs and TJPs [30]. We previously reported the post-transcriptional regulation of VE-cadherin by miR-101 and permeability in hBMVECs exposed to HIV-1 Tat C protein [22]. Increased tyrosine phosphorylation of VE-cadherin and associated catenins are known to compromises the junctional architecture of endothelial cells, which disrupts their physical interactions at cell-cell junctions [8,19,20,31]. Inflammatory conditions change the magnitude of tyrosine phosphorylation of AJPs [19,32-34]. Previously, phosphorylation of Y731 amino acid position in VE-cadherin has been reported to be responsible for impairing the association of VE-cadherin with p120-catenin and β-catenin [15] during leukocyte extravasation [35].

Different mechanisms and pathways have been reported for the regulation of endothelial permeability. Involvement of PPAR/lipid raft-dependent MMP-2 and MMP-3 activation lead to the degradation of occludin and tricellulin, which has been reported in C12-HSL-induced perturbations of epithelial barrier functions [36]. Role of activation of ERK1/2 and Akt signaling has been described for increased BBB permeability through decreased tight junction (TJ) protein expressions in brain microvascular endothelial cells. Tat mediated effects can be attenuated by application of PPAR antagonist [37]. Another *in vitro* BBB model study showed that HIV-1 infection of pericytes can also result into compromised BBB integrity [38]. Another recent study highlighted the role of Rho signaling and CREB in triggering the nuclear localization of ZO-1 and perturbing the endothelial permeability after HIV-1 Tat exposure [39].

In our study, we observed a dose-dependent increase in tyrosine phosphorylation of VE-cadherin at p-Y731 position and β-catenin at p-Y654 position (Figure 1A-E). The Tyr-731 site is considered unique to VE-cadherin in reference to endothelial permeability [15]. VE-cadherin and β-catenin have shown induced phosphorylation at their tyrosine positions accompanied by reduced expression of respective phosphatases VE-PTP and SHP2. Tyrosine phosphorylation of VE-cadherin and β-catenin which disrupts their physical interactions explains increased endothelial permeability and leakage [40].

We studied the role of kinases and phosphatases in HIV-1 Tat induced tyrosine phosphorylation of AJPs. We focused on PYK2 kinase because PYK2 is highly redox sensitive kinase and its signaling gets affected by intracellular ROS levels [26]. PYK2 has been described as a critical regulator of endothelial inflammation via phosphorylating IKK-RelA/p65 pathway, therefore facilitating the nuclear translocation of the RelA/p65 [41]. In our study, increased generation of ROS was observed with the increasing doses of HIV-1 Tat C exposure (Figure 4D). Role of NOX2 in HIV-1 Tat induced generation of ROS has been reported in human astrocytes, where enhanced monocyte adhesion was regulated by ROS mediated increase in ICAM/VCAM expression [42]. Secretion of TNF-α has also been reported to induce NADPH oxidase-derived ROS generation and PYK2 activation in H9c2 cells [43]. In addition, PYK2-dependent tyrosine phosphorylation has been reported to disrupt the association of zonula occludens-2 with the endothelial tight junctions [44]. Our study showed increased intracellular ROS generation, followed by increase in phosphorylation and activation of PYK2, in HIV-1 Tat exposed hBMVECs (Figure 5A, B). This explains the higher levels of tyrosine phosphorylation in β-catenin and VE-cadherin which will lead to increased endothelial cell permeability (Figure 3C). As expected, ROS scavenging has significantly reduced the activation of PYK2 (Figure 5C, D) even in presence of HIV-1 Tat in hBMVECs. PTPs act together with cellular protein tyrosine kinases and control the phosphorylation and dephosphorylation of substrate proteins and associated signaling [45].

VE-PTP enhances VE-cadherin mediated cell–cell adhesion and plays a major role in maintaining endothelial barrier permeability [10]. The significant downregulation of VE-PTP (p ≤0.05) (Figure 2A, E) and significant decrease in association of VE-PTP with VE-cadherin were observed in HIV-1 Tat C exposed hBMVECs (Figure 2B, F). In a recent report, *Vockel et. al*. (2013) reported the generation of ROS through activation of NADPH oxidases and activation of redox-sensitive tyrosine kinase PYK2 and their effect on cell-cell junctions. They suggested that activation of PYK2 could be essential for VE-cadherin/VE-PTP dissociation [46]. In our study, the downregulation and dissociation of VE-PTP from VE-cadherin complex explains the high level of tyrosine phosphorylation of VE-cadherin in HIV-1 Tat treated hBMVECs. Another, non-receptor cytosolic tyrosine phosphatase SHP2 has been reported to be associated with β-catenin [12] and dephosphorylates β-catenin and VE-cadherin in quiscent cells. We observed the significant downregulation of SHP2 in HIV-1 Tat treated hBMVECs in dose dependent manner (Figure 2C) and a significant dissociation of SHP2 from VE-cadherin complex.

Pro-inflammatory insults affect the binding of VE-cadherin and β-catenin [12]. We observed the separation of β-catenin from VE-cadherin complex in pull down experiment in HIV-1 Tat exposed hBMVECs (Figure 3A). The disaggregation of VE-cadherin and β-catenin is most likely due to the increased level of their tyrosine phosphorylation. The disassembly of these proteins led to the enhanced endothelial permeability in HIV-1 Tat treated hBMVECs.ROS generation activated the tyrosine kinases and elevated phosphorylation of AJPs and finally disruption of endothelial integrity. We scavenged the ROS by using DPI followed by HIV-1 Tat treatment. ROS scavenging significantly abolished the PYK2 activation (Figure 5C), sustained SHP2 expression level (Figure 6A), and reduced tyrosine phosphorylation of β-catenin (Figure 6C) in the hBMVECs. These changes resulted into improved permeability in DPI treated hBMVECs (Figure 6E).

We checked the tyrosine phosphorylation of β-catenin in hBMVECs treated with PYK2 inhibiter (PF-431396). PF-431396 abrogates the kinase activity of PYK2/FAK family kinases. A significant decrease in p-Y-β-catenin was observed in the hBMVECs treated with PF-431396 (Figure 7A, B). Our data are in accordance with the previous reports; where β-catenin has been reported to act as a substrate of PYK2 kinase [17]. Phosphorylation of β-catenin was also checked in the hBMVECs, treated with PF-431396 followed by similar amount of HIV-1 Tat protein exposure. The effect of HIV-1 Tat on tyrosine phosphorylation of β-catenin was significantly reduced in hBMVECs treated with PF-431396 as well as in ROS scavenger treated hBMVECs, which suggested that the β-catenin phosphorylation can be controlled by manipulating PYK2 kinase activity and well as intracellular ROS levels. Pharmacological inhibition of PYK2 has been reported as a potential therapeutic strategy to combat acute lung injury in patients as well as mice model of asthma [41,47,48]. PYK2 inhibition rescued the permeability of hBMVECs exposed to HIV-1 Tat protein (Figure 7C). Inhibition of the PYK2 activity by PF-431396 had a partial effect on tyrosine phosphorylation of VE-cadherin (data not shown). This suggested that the phosphorylation events of VE-cadherin are also partially regulated through Src and Rac mediated kinase cascades [20].

## Conclusion

**Figure 8:**
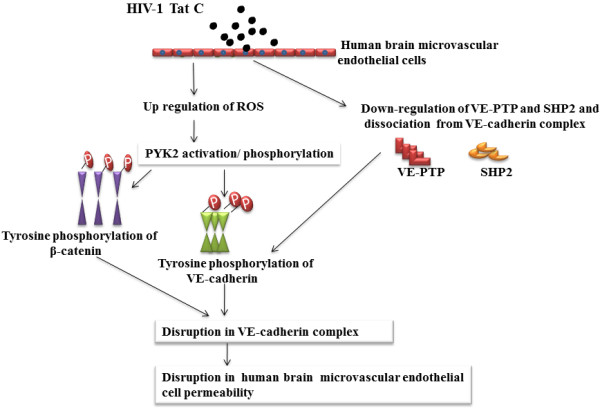
**HIV-1 Tat C mediated tyrosine phosphorylation of VE-cadherin complex and increase in permeability of hBMVECs.** HIV-1 Tat C treatment induces the intracellular ROS generation by activating NADPH oxidases (NOX2/NOX4) proteins in hBMVECs. Elevated intracellular levels of ROS activate the levels of redox sensitive kinase PYK2, which led to increased tyrosine phosphorylation of β-catenin and VE-cadherin. HIV-1 Tat C treatment also downregulates major phosphatases VE-PTP and SHP2 and leads to their dissociation from VE-cadherin complex. These shift in PTK/ PTP ratio in hBMVECs after HIV-1 Tat C exposure leads to destabilized junctional assembly and thereby enhanced endothelial permeability.

HIV-1 Tat C mediated activation of NADPH oxidases (NOX2/ NOX4) led to the increased generation of ROS in hBMVECs and activation of PYK2 kinase. Upregulation of PYK2 activity resulted into the increased tyrosine phosphorylated forms of β-catenin and VE-cadherin. The dissociation of VE-PTP and SHP2 from VE-cadherin complex increased the tyrosine phosphorylation of VE-cadherin/β-catenin, which led to disrupted physical association of AJPs within VE-cadherin complex at endothelial junctions. These conformational alterations increased the permeability in hBMVECs. ROS scavenging as well as inhibition of the PYK2 activity showed a significant potential in combating the HIV-1 Tat C mediated disruption of endothelial permeability (Figure 8). This study suggests the involvement of post-translational modifications of AJPs in the disruption of barrier integrity in hBMVECs by HIV-1 Tat C protein. This finding will be helpful in understanding the effect of viral proteins in a bystander fashion on human brain microvascular endothelial cells.

## Competing interests

All authors declare that they have no competing interests.

## Authors’ contributions

RM and SKS designed research; RM performed research; RM and SKS analyzed data; SKS wrote the paper. Both authors read and approved the final manuscript.

## Authors’ information

RM is currently working as CSIR-Senior Research Fellow and pursuing her Ph.D at CCMB, Hyderabad. SKS is leading a research group in the area of Neurovirology at CCMB, Hyderabad.
